# Age and insulin-like growth factor-1 impact PCNA monoubiquitination in UVB-irradiated human skin

**DOI:** 10.1016/j.jbc.2021.100570

**Published:** 2021-03-19

**Authors:** Rebekah J. Hutcherson, Ryan D. Gabbard, Amber J. Castellanos, Jeffrey B. Travers, Michael G. Kemp

**Affiliations:** 1 Department of Pharmacology and Toxicology, Wright State University Boonshoft School of Medicine, Dayton, Ohio, USA; 2 Department of Dermatology, Wright State University Boonshoft School of Medicine, Dayton, Ohio, USA; 3 Dayton Veterans Affairs Medical Center, Dayton, Ohio, USA

**Keywords:** UV radiation, translesion synthesis, DNA damage, DNA repair, DNA replication, DNA damage response, proliferating cell nuclear antigen (PCNA), ubiquitin, skin, cancer, DDT, DNA damage tolerance, HKGS, human keratinocyte growth supplement, IGF-1, insulin-like growth factor-1, MED, minimal erythemal dose, NMSC, nonmelanoma skin cancer, PCNA, proliferating cell nuclear antigen, TLS, translesion synthesis, IGF-1R, IGF-1 receptor, IGF-1Ri, IGF-1 receptor inhibitor, PCNA-ub, monoubiquitinated PCNA

## Abstract

Nonmelanoma skin cancers occur primarily in individuals over the age of 60 and are characterized by an abundance of ultraviolet (UV) signature mutations in keratinocyte DNA. Though geriatric skin removes UV photoproducts from DNA less efficiently than young adult skin, it is not known whether the utilization of other prosurvival but potentially mutagenic DNA damage tolerance systems such as translesion synthesis (TLS) is altered in older individuals. Using monoubiquitination of the replicative DNA polymerase clamp protein PCNA (proliferating cell nuclear antigen) as a biochemical marker of TLS pathway activation, we find that UVB exposure of the skin of individuals over the age of 65 results in a higher level of PCNA monoubiquitination than in the skin of young adults. Furthermore, based on previous reports showing a role for deficient insulin-like growth factor-1 (IGF-1) signaling in altered UVB DNA damage responses in geriatric human skin, we find that both pharmacological inhibition of the IGF-1 receptor (IGF-1R) and deprivation of IGF-1 potentiate UVB-induced PCNA monoubiquitination in both human skin *ex vivo* and keratinocytes *in vitro*. Interestingly, though the TLS DNA polymerase Pol eta can accurately replicate the major photoproducts induced in DNA by UV radiation, we find that it fails to accumulate on chromatin in the absence of IGF-1R signaling and that this phenotype is correlated with increased mutagenesis in keratinocytes *in vitro*. Thus, altered IGF-1/IGF-1R signaling in geriatric skin may predispose epidermal keratinocytes to carry out a more mutagenic form of DNA synthesis following UVB exposure.

Nonmelanoma skin cancer (NMSC) is the most common neoplasm in humans and is primarily caused by exposures to UV wavelengths of sunlight, which induce potentially mutagenic photoproducts in DNA (1) if not efficiently repaired by the nucleotide excision repair system (2). Because most skin cancers occur in individuals over the age of 60, advanced age is considered a second major risk factor for developing NMSCs (3). Several reports have shown that geriatric skin exhibits a slower rate of UV photoproduct removal than the skin of young adults (4, 5, 6, 7), which indicates that impaired excision repair may contribute to mutagenesis and ultimately carcinogenesis in older individuals.

How the physiological microenvironment of geriatric skin impacts NMSC development in older individuals remains to be fully characterized. Fibroblasts that have undergone replication- or stress-induced senescence *in vitro* are known to express reduced levels of IGF-1 expression in cultured fibroblasts *in vitro* (7, 8). Geriatric skin contains increased numbers of senescent dermal fibroblasts (9) and lower levels of IGF-1 expression and IGF-1 receptor (IGF-1R) activation in epidermal keratinocytes (7). Because keratinocytes express IGF-1Rs but not IGF-1 (10), they are thought to be dependent on the supply from dermal fibroblasts, and several studies with keratinocytes *in vitro*, skin explants *ex vivo*, skin xenografts *in vivo*, and human subjects all support the idea that IGF-1 is important in how keratinocytes respond to UVB radiation (7, 11, 12, 13).

Though geriatric skin exhibits defects in UVB photoproduct removal, there is no evidence that the skin of older individual is more prone to erythema or sunburn. Thus, geriatric skin may utilize DNA damage tolerance (DDT) pathways or other DNA damage responses to a greater extent than young adult skin. These DDT pathways encompass a variety of processes that take place at replication forks stalled at UVB photoproducts, including translesion synthesis (TLS), template switching, and repriming (14, 15). Though significant effort has been placed on understanding these processes at the molecular level *in vitro*, little work has been done to determine how these pathways are utilized in human skin. This dearth of information is due in part to the lack of biochemical markers of these processes that can be exploited for use in skin tissue.

One DDT pathway that is well recognized to impact UV-induced skin carcinogenesis is TLS, which involves the recruitment of specialized DNA polymerases to damaged DNA that can insert nucleotide opposite DNA lesions in either an error-free or an error-prone manner (15). The variant form of the skin cancer-prone disease xeroderma pigmentosum is due to mutations in the TLS DNA polymerase Pol eta (16, 17, 18), which is capable of accurately replicating UV-induced cyclobutane pyrimidine dimers (18, 19, 20). In the absence of pol eta function, other TLS polymerases are recruited to replicate damaged DNA in a more error-prone manner. Though it is not fully clear how or why specific TLS polymerases are recruited to UV lesions in DNA, the monoubiquitination of the replicative polymerase clamp protein PCNA (proliferating cell nuclear antigen) is generally considered to be a key signal involved in the recruitment of TLS polymerases to sites of damage because these enzymes contain ubiquitin-binding domains (15, 17).

Here we show that an antibody against the monoubiquitinated form of PCNA can be used to readily detect this important biochemical marker of TLS in UVB-irradiated human skin. We further explore the use of this new assay to explore how subject age and IGF-1 signaling impact the UVB-dependent induction of PCNA monoubiquitination in the skin. Thus, this work sheds new light on how the cellular DNA damage response may be altered in IGF-1-deficient geriatric skin to contribute to mutagenesis and NMSC development.

## Results

### PCNA monoubiquitination can be detected in UVB-irradiated human skin

**Figure 1:**
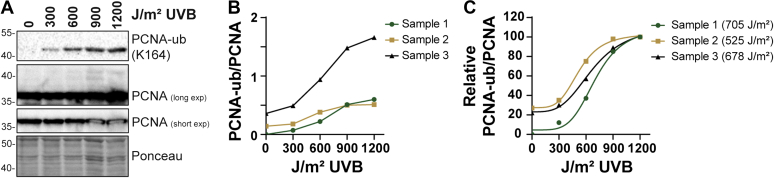
**PCNA monoubiquitination can be detected in human skin epidermis.***A*, immunoblot analysis of epidermal protein from human skin exposed to the indicated fluences of UVB radiation. The membrane was stained with Ponceau, probed with an antibody against the monoubiquitinated form of PCNA (monoubiquitinated PCNA [PCNA-ub]; K164), and then reprobed to detect the unmodified form of PCNA. *B*, densitometry was used to quantify the levels of monoubiquitinated and unmodified PCNA from UVB-irradiated skin samples from three different individuals. The PCNA-ub/PCNA ratio was calculated and plotted. *C*, the PCNA-ub/PCNA ratios in B were normalized to the 1200 J/m^2^ treatment for each skin sample, which was set to an arbitrary value of 100. A variable slope, four-parameter linear regression was then used to calculate the UVB dose resulting in a half maximal response.

Activation of the TLS pathway is routinely assayed in cultured cells *in vitro* by immunoblotting cell or chromatin fractions with an anti-PCNA antibody that can detect both the unmodified protein and the monoubiquitinated form of PCNA that is shifted by ∼8-kDa shift on SDS gels (17). To determine whether this modification can be observed in human skin tissue, we exposed human skin samples discarded during routine panniculectomies and other surgeries to increasing fluences of UVB radiation. Following a short incubation, epidermal protein lysates were prepared and analyzed by immunoblotting. As shown in Figure 1*A*, the ∼35-kDa unmodified form of PCNA could be readily detected with the routinely employed anti-PCNA PC10 monoclonal antibody. However, we were unable to readily or reproducibly detect the shifted, monoubiquitinated form with this antibody. In contrast, when the blot was probed with a monoclonal antibody that specifically recognizes the ubiquitinated form of PCNA, a clear band at ∼43-kDa that displayed a strong correlation with UVB dose could be observed. We note that to our knowledge, this is the first demonstration of PCNA monoubiquitination in any organ tissue.

Further analysis of monoubiquitinated PCNA (hereby referred to as PCNA-ub) and unmodified PCNA from several different skin donors showed there to be interindividual variability in both basal PCNA-ub and the magnitude of the response to UVB. Nonetheless, a clear UVB dose-dependent increase in PCNA-ub was observed in all three samples that were tested (Fig. 1*B*). By normalizing the PCNA-ub/PCNA signals to the high dose of 1200 J/m^2^ UVB sample and performing nonlinear regressions, UVB doses yielding a half-maximal response ranged from 500 to 700 J/m^2^. These UVB doses correspond to approximately twice a minimal erythemal doses (MED) for the lightly pigmented Fitzpatrick Type I-II skin used in these studies (21).

### Geriatric skin displays elevated PCNA monoubiquitination following UVB exposure

**Figure 2:**
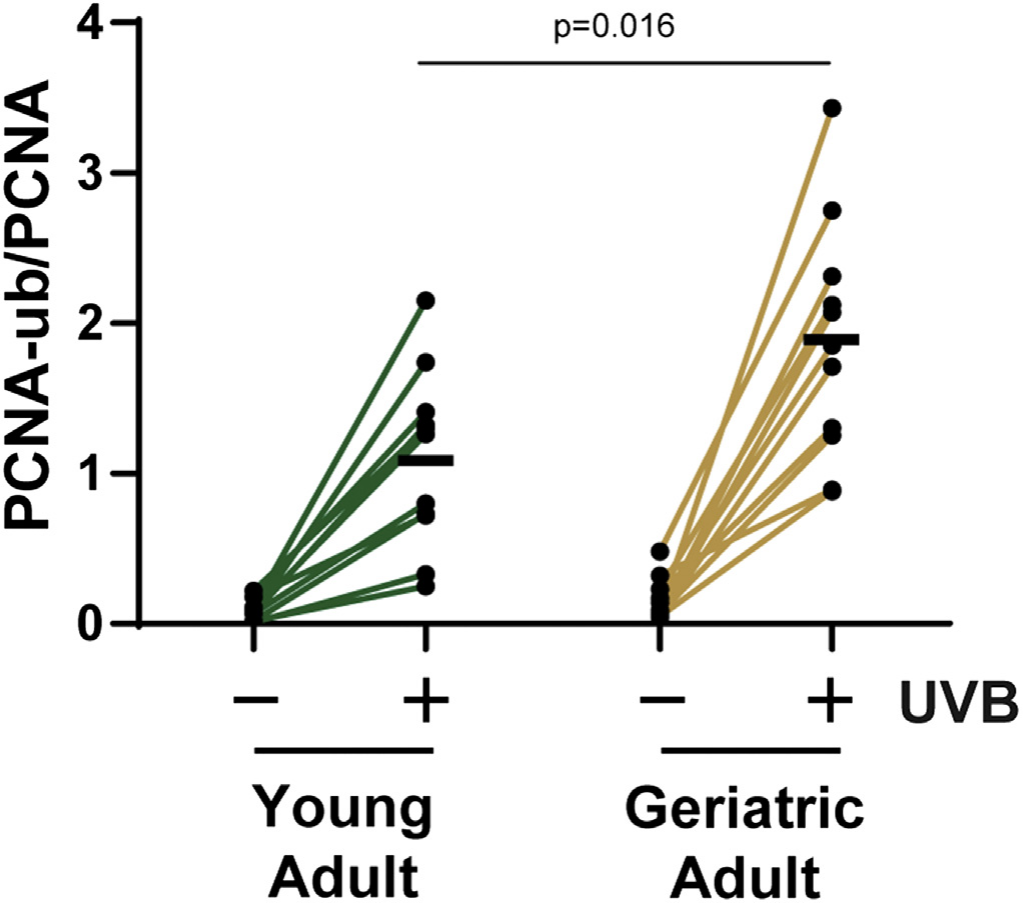
**Geriatric skin exhibits higher levels of PCNA monoubiquitination following UVB exposure than young adult skin.** Two 5 mm punch biopsies were obtained from the buttock skin of young adults (21–30 years of age) and geriatric adults (over age 65) (n = 11 for each group), exposed to 700 J/m^2^ UVB radiation (or *left* nonirradiated), and then incubated in a 37 °C water bath for 2.5 h. Epidermal protein was analyzed for PCNA monoubiquitination as described in Figure 1. The PCNA-ub/PCNA ratio was calculated and plotted for each donor. The *black bar* indicates the average response for each of the UVB-treated samples. An unpaired *t*-test was used to compare the responses in the UVB-irradiated young adult and geriatric adult skin samples.

With this assay in hand, we next sought to determine how age impacts PCNA monoubiquitination in UVB-irradiated human skin. We therefore recruited young adults 21–30 years of age and geriatric adults over the age of 65 and obtained skin punch biopsies from buttock skin. One of the biopsies was exposed to 700 J/m^2^ UVB and incubated for 2.5 h. Epidermal protein was then analyzed by immunoblotting with PCNA-ub and PCNA antibodies, and the PCNA-ub/PCNA signal ratios were calculated. As shown in Figure 2, UV induced an increase in PCNA-ub in each of the skin samples. Interestingly, though there is significant interindividual variability (4–8-fold) in PCNA-ub levels following UVB exposure, the level of PCNA-ub was found to be increased by approximately 70% on average in geriatric subject skin in comparison to young adult skin. These results indicate that geriatric skin may utilize the TLS pathway of DNA replication to a greater degree than young adult skin, potentially to deal with the slower rate of UV photoproduct removal (4, 5, 6, 7).

### Inhibition of IGF-1 receptor signaling potentiates UVB-dependent induction of PCNA monoubiquitination in human skin *ex vivo*

**Figure 3:**
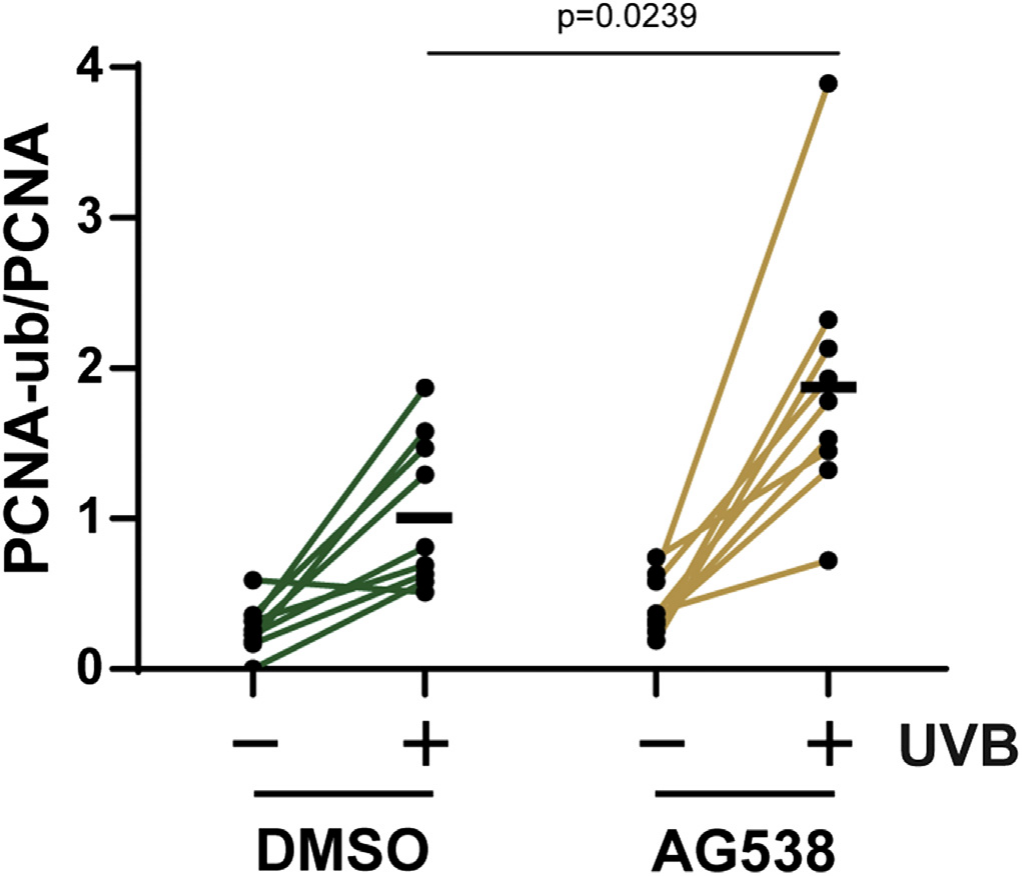
**IGF-1 receptor inhibition potentiates PCNA monoubiquitination in UVB-irradiated human skin explants *ex vivo*.** Human skin samples (n = 9) were treated topically with vehicle (DMSO) or 20 μM AG538 for 30 min before exposure to 700–800 J/m^2^ UVB. Skin biopsies were obtained 1–2 h later, and then PCNA monoubiquitination was measured and quantified as in Figures 1 and 2, respectively. The *black bar* indicates the average response for each of the UVB-treated samples, and an unpaired *t*-test was used to compare the responses in the UVB-irradiated DMSO- and AG538-treated skin samples.

Geriatric skin exhibits numerous changes in both structure and function that may impact how epidermal cells respond to UVB radiation (22, 23, 24, 25). One factor that has been demonstrated to impact several UVB responses in geriatric skin is the expression of IGF-1, which, due to increased fibroblast senescence, is expressed at lower levels in geriatric skin (7). To determine whether IGF-1 impacts PCNA monoubiquitination in human skin epidermis, human skin explants were treated topically with either vehicle (DMSO) or the IGF-1 receptor inhibitor (IGF-1Ri) AG538, which has previously been shown to serve as a convenient *ex vivo* model that mimics the loss of IGF-1 signaling in geriatric skin (13). Skin explants from nine different middle-aged donors were then exposed to UVB radiation and analyzed for epidermal PCNA-ub levels. As shown in Figure 3, a UVB-dependent increase in PCNA-ub was observed in nearly all of the skin explant samples. Moreover, inhibiting the IGF-1R generally led to a nearly twofold higher level of PCNA-ub than in DMSO-treated skin following UVB irradiation. Thus, these results support the data in Figure 1 and indicate that the lack of IGF-1 signaling may result in a greater reliance on the TLS pathway in UVB-exposed skin.

### The loss of IGF-1 receptor signaling potentiates UVB-induced PCNA monoubiquitination in cultured keratinocytes *in vitro*

**Figure 4:**
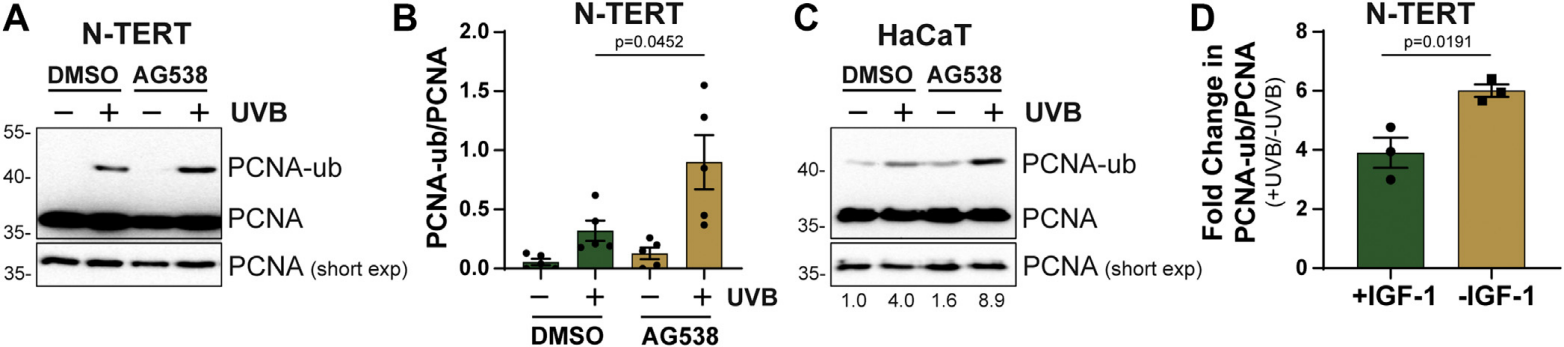
**Loss of IGF-1 receptor signaling potentiates PCNA monoubiquitination in UVB-keratinocytes *in vitro*.***A*, Telomerase-immortalized N-TERT keratinocytes were treated with vehicle (DMSO) or 10 μM AG538 for 30 min before exposure to 50 J/m^2^ UVB radiation. Cells were harvested 30 min later and fractionated to enrich for chromatin-associated proteins, which were analyzed by immunoblotting. Short and long exposures of the blot are shown. *B*, the ratio of PCNA-ub to PCNA from five independent experiments was calculated for each experiment and then blotted with the average response. Error bars indicate the SEM. *C*, HaCaT keratinocytes were treated as described in A. Quantitation of relative the PCNA-ub/PCNA ration is shown below the blot. *D*, N-TERTs were cultured in medium lacking or containing IGF-1 for 24 h prior to UVB exposure and processing as in A. The fold change in PCNA-ub/PCNA was calculated from three independent experiments.

To extend our findings with human skin to cultured keratinocytes *in vitro*, telomerase-immortalized N-TERT keratinocytes were pretreated with DMSO or the IGF-1Ri AG538 and then exposed to a low dose of UVB radiation. As shown in Figure 4*A*, immunoblot analysis of chromatin-enriched cell fractions demonstrated that UVB treatment induced PCNA monoubiquitination but to a greater extent in cells treated with the IGF-1Ri. Quantitation from several independent experiments confirmed these findings and showed PCNA-ub levels to be 2–3-fold higher in cells with inactive IGF-1 Rs (Fig. 4*B*). Similar results were observed using HaCaT keratinocytes (Fig. 4*C*). Lastly, inhibiting IGF-1R signaling *via* deprivation of IGF-1 from the cell culture medium also potentiated PCNA monoubiquitination in UVB-irradiated N-TERTs (Fig. 4*D*). Together, these results demonstrate that modulating IGF-1/IGF-1R signaling in keratinocytes *in vitro* results in a similar response to UVB radiation as total skin epidermal cells *ex vivo*.

### Loss of IGF-1 signaling is associated with mutagenesis and a failure of pol eta to accumulate on UVB-damaged chromatin

**Figure 5:**
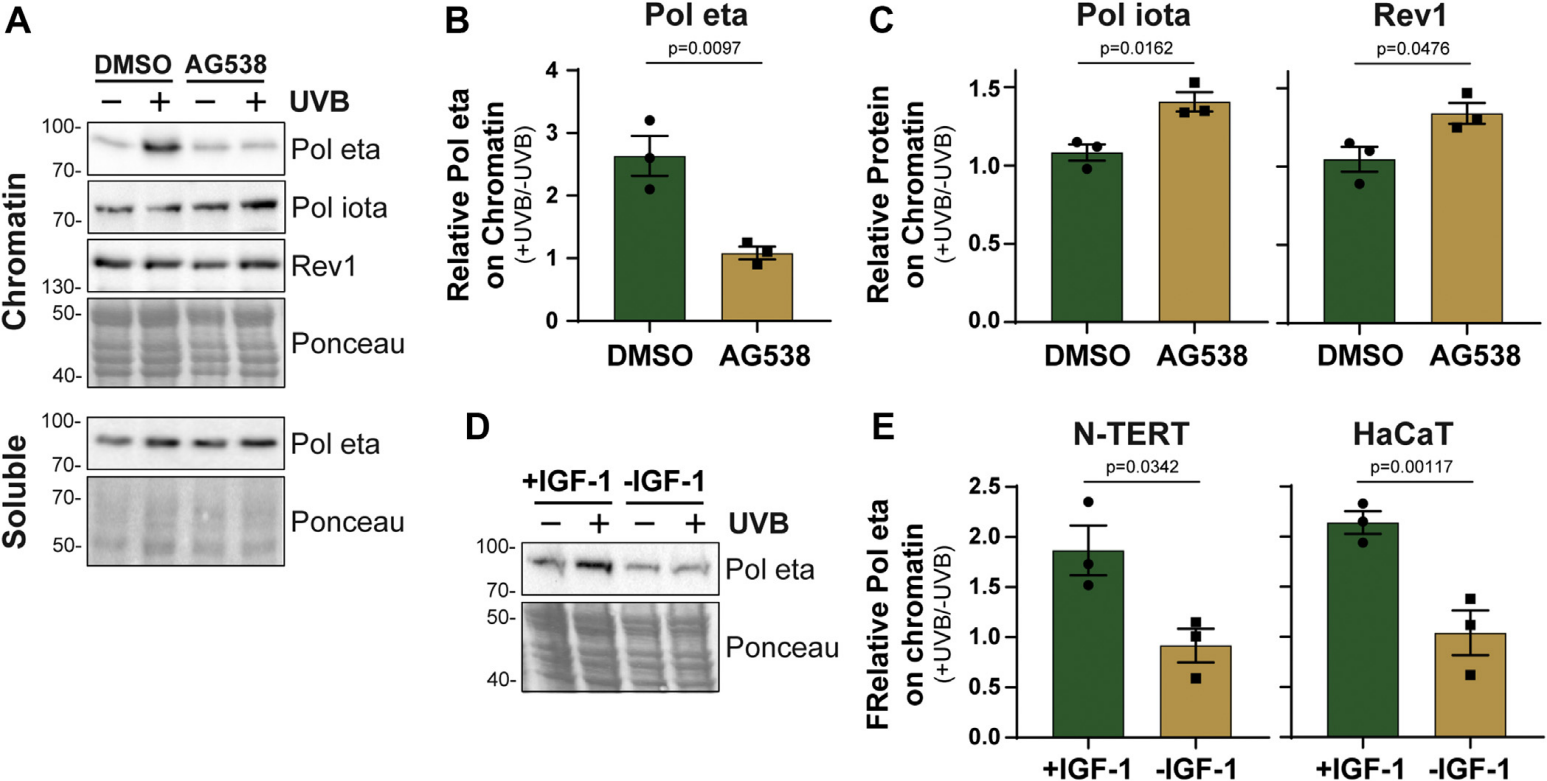
**Loss of IGF-1 signaling is associated with a defect in Pol eta accumulation on UVB-damaged chromatin.***A*, Chromatin lysates from N-TERTs treated with DMSO or AG538 and exposed to UVB as in Figure 4*A* were analyzed by immunoblotting. *B* and *C*, the fold change in TLS polymerase level on chromatin was calculated and graphed from three independent experiments. *D*, Soluble lysates from N-TERTs were examined by immunoblotting. *E*, N-TERTs grown in the absence or presence of IGF-1 for 24 h were exposed to 50 J/m^2^ UVB, incubated for 30 min, and then chromatin fractions were analyzed for Pol eta protein levels. *F*, the fold change in chromatin-associated Pol eta levels was calculated in three independent experiments performed as in Figure 6*C* with N-TERTs and HaCaT cells.

The monoubiquitination of PCNA serves as a signal for the recruitment of specialized TLS polymerases to damaged DNA (15, 17). In the case of UVB-induced DNA damage, the TLS polymerase Pol eta is capable of accurately replicating the common thymine dimers induced by UV radiation. In response to UVC radiation, Pol eta has been found to accumulate on chromatin in cultured cells *in vitro*, which can be assayed by immunoblotting chromatin-enriched cell fractions (26). As shown in Figure 5, *A* and *B*, we observed that Pol eta was enriched by 2–3-fold on chromatin within 30 min of exposure of N-TERTs to a low dose of UVB radiation. However, this enrichment was nearly completely abrogated by treatment with the IGF-1Ri AG538. Importantly, IGF-1R inhibition did not affect the amount of Pol eta present in the soluble fraction of the cells (Fig. 5*A*).

In the absence of Pol eta function, other TLS polymerases are likely required to replicate unrepaired UVB photoproducts. Consistent with this hypothesis, we observed that both Pol iota and Rev1, which play recognized roles in UV mutagenesis (15), become modestly enriched on chromatin in UVB-irradiated keratinocytes in which IGF-1 signaling has been inhibited (Fig. 5, *A* and *C*). A similar defect in Pol eta recruitment to chromatin was observed when N-TERTs and HaCaT keratinocytes were deprived of IGF-1 in the culture medium (Fig. 5, *D*, *F* and *E*). Thus, the major TLS polymerase thought to be responsible for accurately replicating unrepaired UV photoproducts fails to associate with UV-damaged chromatin following UVB exposure in cultured keratinocytes *in vitro* when IGF-1 signaling is disrupted.

**Figure 6:**
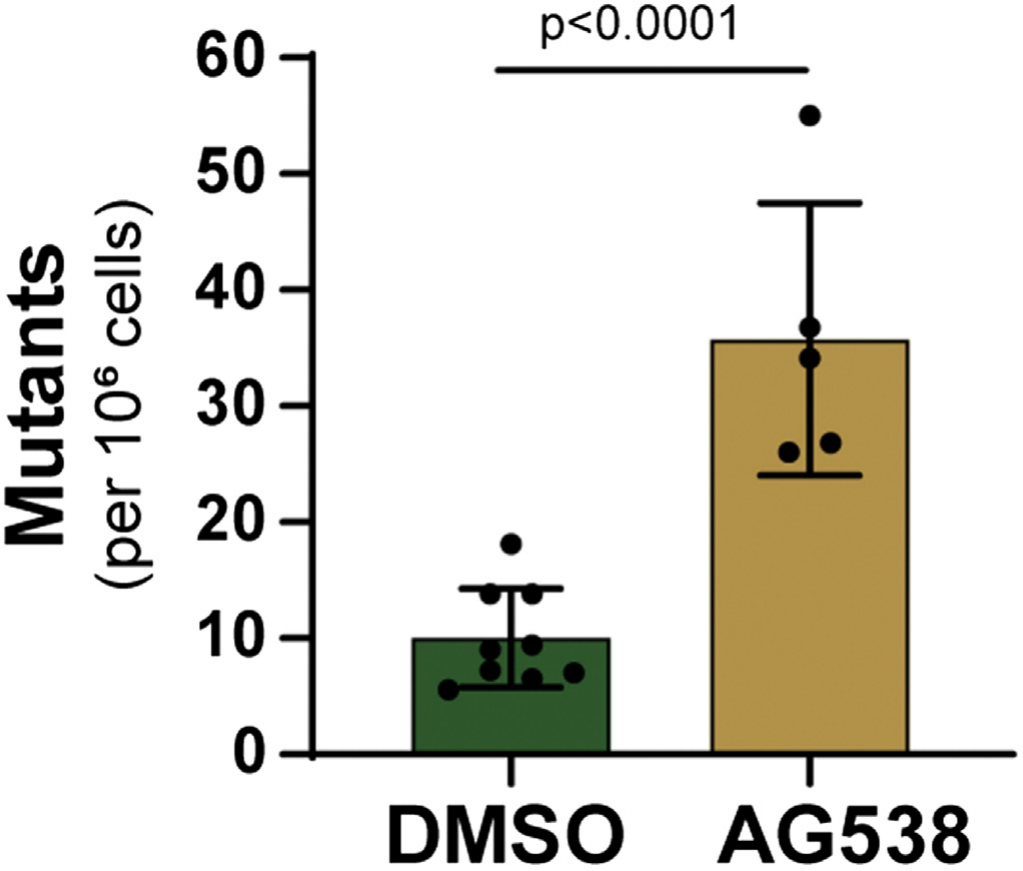
**Loss of IGF-1 signaling is associated increased mutagenesis in UVB-irradiated keratinocytes.** HaCaT cells were treated with DMSO or the IGF-1R inhibitor AG538 as in Figure 4, and media was replaced with drug-free medium 5 h after UVB-exposure. Five days later, cells were replated and selected with 6-thioguanine to detect cells with mutations at the HPRT locus. The graphs show the average (±SEM) number of mutant cells (per million cells plated) along with the individual experimental values, and an unpaired *t*-test was used to analyze the results.

Increased PCNA monoubiquitination coupled with the failure to recruit Pol eta to damaged chromatin suggests that other, potentially more mutagenic DNA polymerases may be involved in replicating unrepaired UV lesions when IGF-1 signaling is lost. To determine whether there is a higher level of mutagenesis under these conditions, we treated UVB-irradiated HaCaT cells with 6-thioguanine to select for cells with mutations at the HPRT locus, which imparts resistance to the drug (27). As shown in Figure 6, HaCaT cells treated with DMSO vehicle exhibited a low mutation frequency of 10 per million cells. In contrast, when cells were treated with the IGF-1Ri AG538, the mutation rate was increased approximately threefold. We conclude from these experiments that the loss of IGF-1 signaling increases mutagenesis induced by UVB radiation.

## Discussion

Though the structure and physiological microenvironment of the skin are known to change over the course of the lifetime (22, 23, 24, 25) and to lead to an increased risk of NMSC development in older individuals (3), the mechanisms responsible for this effect remain to be fully elucidated. Historically, most experimental studies of UVB-irradiated human skin have been restricted to measurements of UVB photoproduct levels at various time points following UVB exposure. Though this information is valuable, additional work is necessary to understand whether the plethora of DNA damage responses that govern the cell fate following UVB exposure are impacted by age and other factors of the skin.

Here we showed for the first time that PCNA monoubiquitination, a posttranslational modification associated with the activation of the TLS pathway and the recruitment of specialized DNA polymerases to sites of DNA damage (15, 17), can be readily detected in human skin epidermis following UVB exposure (Fig. 1). Moreover, the observation that this response is potentiated by advanced age (Fig. 2) and the loss of IGF-1R signaling (Fig. 3) suggest that the age-dependent decline in IGF-1 expression in the skin (7, 12) impacts several canonical DNA damage responses in addition to the rate of removal of UVB photoproducts (13) and the activation of ATR kinase signaling (28).

Moreover, our observation that the TLS polymerase Pol eta is not efficiently recruited to UVB-damaged chromatin in IGF-1-deficient keratinocytes *in vitro* (Fig. 5) suggests that other, potentially more mutagenic DNA polymerases are likely responsible for replicating UVB-damaged DNA under these conditions. Pol eta recruitment to sites of DNA damage has been reported to require both ATR kinase and Protein Kinase C signaling (29, 30, 31), and thus it is possible that the defects in ATR signaling that we (28) and others (32) have reported in IGF-1-deficient keratinocytes *in vitro* contribute to the aberrant recruitment. Precisely how IGF-1/IGF-1R signaling impacts PCNA ubiquitination and TLS polymerase recruitment remains to be better defined. In addition to the regulation of AKT and MAPK signaling (33), which could directly or indirectly impact various aspects of cell metabolism, recent studies indicate that the IGF-1R can directly interact with and phosphorylate PCNA in some cancer cell lines (34, 35). Thus, additional work is necessary to understand how the IGF-1R impacts genome stability in UVB-irradiated keratinocytes *in vitro* and within the context of human skin.

We would also note that we recently showed that Pol eta fails to be properly induced at the transcriptional level following UVB exposure in human keratinocytes *in vitro* and skin *ex vivo* when IGF-1 signaling is abrogated (12). Together, these results suggest that chronically sun-exposed regions of geriatric skin may be particularly vulnerable to mutagenic forms of DNA synthesis due to an increased reliance on TLS *via* elevated PCNA monoubiquitination and an inability to recruit Pol eta to sites of DNA damage. However, future studies will be needed to test this hypothesis. Lastly, because dermal wounding modes of skin rejuvenation have been shown to restore IGF-1 expression in geriatric skin (9, 36, 37), it will be important to determine whether these clinical interventions improve the ability of geriatric skin to accurately replicate unrepaired UVB photoproducts.

## Experimental procedures

### Human skin samples

Experiments with human skin involved the skin obtained from two different sources. In the first study, two 5 mm skin punch biopsies were obtained from each of 11 young adults (21–30 years of age) and 11 geriatric adults (over 65 years of age) with Fitzpatrick Type I-II skin. This human subject research protocol was approved by the Wright State University Institutional Review Board and abides by the Declaration of Helsinki principles. Within 30 min of excision, one biopsy was then exposed to 700 J/m^2^ UVB with a Philips F20T12 UVB bulb at a dose rate of 5 J/m^2^/sec. The other biopsy was sham-treated, and then both biopsies were incubated for 2.5 h incubation in a 37 °C water bath before freezing in liquid nitrogen. Epidermal lysates were prepared in RIPA buffer containing protease and phosphatase inhibitors and then 3 μg of each sample was separated by SDS-PAGE and analyzed by immunoblotting. In the second set of experiments, discarded, deidentified skin from panniculectomies and other surgical procedures was treated topically with DMSO vehicle or 20 μM AG538 for 30 min before exposure to UVB radiation and incubation for 1–2 h in a water bath. Skin punch biopsies (5–6 mm) were then obtained and snap frozen in liquid nitrogen.

### Cell culture

N-TERT cells were cultured as previously described in EpiLife medium containing human keratinocyte growth supplement (HKGS) and penicillin/streptomycin (28). HaCaT cells were usually grown in DMEM containing FBS and penicillin/streptomycin, but HaCaT cells were also adapted for growth in EpiLife medium in certain experiments in which cells were cultured in EpiLife medium with HKGS lacking or containing IGF-1. DMSO or the IGF-1R inhibitor AG538 was added to medium as previously described (28). Cells were irradiated with the same UVB light source as described for human skin.

### Immunoblotting

To prepare skin epidermal samples for immunoblot analysis, the punch biopsies were briefly heated in a water bath at 60–70 °C for 6 s and then placed in an ice bath for 9 s. A curette was then used to separate the dermis from the epidermis, which was then sonicated in RIPA buffer and centrifuged for 20 min at maximum speed in a microcentrifuge at 4 °C. To prepare protein lysates from cultured keratinocytes, cells were extracted twice with a modified cytoskeletal buffer (10 mM Tris-HCl (pH 7.4), 100 mM NaCl, 3 mM MgCl2, 1 mM EDTA, 1 mM Na3VO4, 10 mM NaF, and 0.1% Triton X-100). The soluble or chromatin-enriched fractions were then used for immunoblotting as described in the figure legend. Cell and epidermal lysates were separated by SDS-PAGE, transferred to a nitrocellulose membrane, and then stained with Ponceau S to ensure equal protein loading. Following washing with TBST (Tris-buffered saline containing 0.1% Tween-20) and blocking in 5% milk in TBST, blots were probed with 1:2000 or 1:5000 dilutions of antibodies against ubiquityl-PCNA (Lys 164; Cell Signaling #13439), PCNA (Santa Cruz sc-56), Pol eta (Santa Cruz sc-17770), Pol iota (GeneTex GTX112137), and Rev1 (Santa Cruz sc-393022). After washing, the blots were probed with HRP-coupled anti-mouse or anti-rabbit IgG (ThermoFisher) secondary antibodies for 1–2 h at room temperature. Chemiluminescence was visualized with either Clarity Western ECL substrate (Bio-Rad) or SuperSignal West Femto substrate (Thermo Scientific) using a Molecular Imager Chemi-Doc XRS+ imaging system (Bio-Rad). Signals in the linear range of detection were quantified by densitometry using Image Lab (Bio-Rad) and normalized as previously described (38) or calculated as fold changes in UVB-irradiated samples relative to the nonirradiated sample in each experiment. Unpaired *t*-tests were used to compare the UVB-dependent changes in protein levels between the treatment groups.

### HPRT mutagenesis assays

HaCaT cells were pretreated with DMSO or the IGF-1R inhibitor AG538 for 30 min before exposure to 50 J/m^2^ UVB. Media was replaced with drug-free medium 5 h later. Following a 5 days of cell proliferation, cells were replated for analysis of mutagenesis at the HPRT locus by selection with 4 μg/ml 6-thioguanine for 2 weeks (27). Surviving colonies were stained with crystal violet to calculate the mutant frequency. An ANOVA was run to analyze the data sets.

## Data availability

All data are provided in the article and/or are available upon request to mike.kemp@wright.edu

## Conflict of interest

The authors declare that they have no conflicts of interest with the contents of this article.
